# Supplementary material for: Active site specificity profiling datasets of matrix metalloproteinases (MMPs) 1, 2, 3, 7, 8, 9, 12, 13 and 14
Source: Data Brief. 2016 Feb 22;7:299–310. doi: 10.1016/j.dib.2016.02.036 (PMC4777984; doi:10.1016/j.dib.2016.02.036)
Supplement: Supplementary file 10 — Supplementary material [file mmc10.zip › WebPICS_hMMP1_G_1%/subsite_coop.html]

 

PICS results


- # About
- # Manual
- # Analysis
- # Results

- Redraw
- Seqlogo
- Dependency
- Coop

**Potential subsite cooperativity analysis for hMMP1\_G\_1%**   
Minimum difference for subsite dependency was set to +- 10 percentage-points  
Subsite dependency was checked for all positional occurences above 1 x natural abundance  
**Subsite dependency check is restricted to P3 - P3'**

|  |  |  |  |
| --- | --- | --- | --- |
| Fixed residue | Affected residue(s) | Change (percentage-points) | Vice-Versa change |
| P3\_A | P1\_Y | 11.1 | 38.1 |
| P3\_A | P2prime\_L | 12.3 | 18.5 |
| P3\_P | P1\_N | -11.0 | -11.5 |
| P3\_P | P1prime\_I | 12.2 | 12.2 |
| P3\_P | P1prime\_L | -25.2 | -10.1 |
| P3\_P | P2prime\_V | 13.7 | 26.4 |
| P3\_T | P1\_R | 11.1 | 13.5 |
| P3\_T | P1prime\_C | 21.0 | 28.8 |
| P3\_T | P2prime\_T | 10.3 | 11.3 |
| P3\_T | P2prime\_V | 17.0 | 14.4 |
| P3\_V | P1\_A | 10.1 | 14.6 |
| P3\_V | P1\_H | 13.2 | 30.2 |
| P3\_V | P2\_L | 12.3 | 12.3 |
| P3\_V | P2prime\_K | 25.5 | 17.7 |
| P3\_V | P3prime\_K | 35.9 | 57.3 |
| P2\_A | P1prime\_C | 10.4 | 15.5 |
| P2\_A | P2prime\_T | 25.4 | 30.5 |
| P2\_A | P3prime\_T | 16.3 | 17.8 |
| P2\_C | P1\_H | 19.4 | 11.1 |
| P2\_C | P2prime\_A | 40.5 | 13.5 |
| P2\_C | P3prime\_K | 42.1 | 16.8 |
| P2\_F | P1\_N | 23.9 | 13.9 |
| P2\_F | P3prime\_M | 11.1 | 38.9 |
| P2\_F | P3prime\_N | 19.9 | 25.3 |
| P2\_K | P1\_A | 16.3 | 11.9 |
| P2\_K | P2prime\_I | 15.5 | 10.4 |
| P2\_L | P1\_P | 20.2 | 54.0 |
| P2\_L | P2prime\_L | 12.3 | 12.3 |
| P2\_L | P3prime\_K | 17.1 | 27.3 |
| P2\_L | P3prime\_N | 10.1 | 14.6 |
| P2\_L | P3prime\_Q | 10.1 | 14.6 |
| P2\_M | P2prime\_Y | 13.5 | 20.2 |
| P2\_N | P1\_A | 24.6 | 13.4 |
| P2\_N | P1\_C | 12.7 | 15.2 |
| P2\_N | P2prime\_T | 42.1 | 25.2 |
| P2\_Q | P1\_Y | 23.0 | 46.0 |
| P2\_Q | P2prime\_L | 23.0 | 20.1 |
| P2\_Q | P3prime\_Q | 19.9 | 25.3 |
| P2\_Y | P1\_S | 18.2 | 11.6 |
| P2\_Y | P1prime\_C | 10.4 | 15.5 |
| P2\_Y | P1prime\_I | 30.2 | 14.5 |
| P2\_Y | P2prime\_F | 20.2 | 40.5 |
| P2\_Y | P2prime\_V | 14.7 | 13.6 |
| P2\_Y | P2prime\_Y | 13.5 | 40.5 |
| P2\_Y | P3prime\_V | 20.6 | 15.5 |
| P1\_A | P2prime\_F | 13.4 | 24.6 |
| P1\_A | P2prime\_L | 23.7 | 16.3 |
| P1\_A | P3prime\_K | 10.3 | 11.3 |
| P1\_C | P3prime\_A | 44.1 | 11.0 |
| P1\_H | P2prime\_K | 38.8 | 11.8 |
| P1\_H | P2prime\_Q | 10.3 | 14.4 |
| P1\_H | P3prime\_K | 49.2 | 34.4 |
| P1\_H | P3prime\_M | 11.1 | 19.4 |
| P1\_P | P2prime\_V | 23.0 | 10.6 |
| P1\_P | P2prime\_Y | 13.5 | 20.2 |
| P1\_R | P3prime\_N | 13.5 | 11.1 |
| P1\_R | P3prime\_Q | 13.5 | 11.1 |
| P1\_S | P1prime\_C | 14.8 | 34.9 |
| P1\_S | P1prime\_I | -14.5 | -11.1 |
| P1\_S | P2prime\_V | 10.8 | 15.7 |
| P1\_Y | P2prime\_L | 30.2 | 13.2 |
| P1\_Y | P3prime\_M | 25.4 | 44.4 |
| P1\_Y | P3prime\_Q | 34.2 | 21.7 |
| P1prime\_C | P2prime\_T | 29.6 | 23.7 |
| P1prime\_C | P3prime\_T | 16.3 | 11.9 |
| P1prime\_M | P3prime\_T | 41.3 | 22.5 |
| P2prime\_F | P3prime\_V | 37.3 | 14.0 |
| P2prime\_I | P3prime\_N | 24.6 | 26.9 |
| P2prime\_L | P3prime\_K | 10.9 | 17.3 |
| P2prime\_L | P3prime\_Q | 16.3 | 23.7 |
| P2prime\_T | P3prime\_T | 21.3 | 19.4 |
| P2prime\_T | P3prime\_V | 17.3 | 10.9 |
| P2prime\_Y | P3prime\_V | 62.3 | 15.6 |

  
**PICS analysis of protease: hMMP1\_G\_1%**  
126 cleavage sites analyzed  
PICS library made with (T)rypsin, (G)luC or (C)hymotrypsin:   
Cutoff for graphic display: 2 x natural abundance  

|  |  |
| --- | --- |
| Positional occurences  (table for total and relative (in %) values) | Occurences relative to natural abundance (table) |
|  |  |
